# Multilevel analysis of patients with hypophosphatasemia: genomic, epigenomic and isoenzyme activity profiles

**DOI:** 10.3389/fendo.2026.1910750

**Published:** 2026-10-30

**Authors:** Álvaro Del Real, Sofía Álvarez Villalobos, María Ortiz Espejo, Carolina Sañudo, Ana I. Vega, Leyre Riancho-Zarrabeitia, Teresa Amigo, Maria Teresa García-Unzueta, Nuria Puente Ruiz, Alex Garcia Tellado, Olga Gutiérrez Saiz, Javier Lopez Montiel, Juan Ansede-Bermejo, Jose Luis Fernández-Luna, Domingo González-Lamuño, Angel Carracedo, Jose A. Riancho

**Affiliations:** 1 Hospital Universitario Marques de Valdecilla, Santander, Spain; 2 Instituto de Investigacion Marques de Valdecilla, Santander, Spain; 3 Universidad of Cantabria; Faculty of Medicine, Santander, Spain; 4 Hospital General Universitario Sierrallana., Torrelavega, Spain; 5 Centro de Investigación Biomédica en Red de Enfermedades Raras, Madrid, Spain; 6 Reference Laboratory of Catalonia, NGS, L’Hospitalet de Llobregat, Spain; 7 Universidade de Santiago de Compostela Centro Singular de Investigacion en Medicina Molecular e Enfermidades Cronicas, Santiago de Compostela, Spain; 8 Fundación Pública Galega Medicina Xenómica, Santiago de Compostela, Spain

**Keywords:** alkaline phosphatase, ALPL, bone-specific alkaline phosphatase, DNA methylation, hypophosphatasia, isoenzymes

## Abstract

**Background:**

Hypophosphatasemia is the biochemical hallmark of (HPP), a rare genetic condition caused by deficient activity of tissue-nonspecific alkaline phosphatase (ALP) due to pathogenic variants in the *ALPL* gene. However, a substantial number of patients with persistently low serum levels of ALP (hypophosphatasemia) lack identifiable mutations, complicating diagnosis and management.

**Aim:**

To investigate the molecular basis of low ALP activity in adult patients without detectable *ALPL* mutations using an integrative multi-omics approach and isoenzyme profiling.

**Methods:**

We studied 50 adult patients with low serum ALP and 20 healthy controls. Genetic analysis included targeted sequencing of *ALPL* exons and promoter, and whole-exome sequencing in mutation-negative cases. Promoter methylation was explored by MassARRAY, and *ALPL* mRNA expression was determined by RT-qPCR. Total and bone-specific ALP, and bone turnover markers were determined. Isoenzyme fractions were analyzed by agarose gel electrophoresis.

**Results:**

Pathogenic *ALPL* variants were identified in 24 patients, whereas the remaining 26 were non-carriers. Non-carriers did not have identifiable variants in the *ALPL* promoter or in other genes related to bone and mineral homeostasis. Likewise, *ALPL* expression levels, and promoter methylation were similar in all groups. PINP and β-CTX levels were similarly reduced in carriers and non-carriers, in comparison with controls. In contrast, mutation carriers exhibited a marked reduction in the hepatic ALP isoenzyme, while mutation-negative individuals showed relatively preserved liver ALP activity. Moreover, in non-carriers, we did not find the expected correlation between total and bone-specific ALP in serum.

**Conclusions:**

Taken together, these findings, along the normal serum levels of pyridoxal-phosphate, suggest that in patients with persistent hypophosphatasemia without *ALPL* mutations, low serum ALP levels may reflect an enzymatic defect affecting bone more than liver. Longitudinal follow-up of these patients will be essential to determine whether low ALP levels are associated with long-term musculoskeletal consequences.

## 1 Introduction

Hypophosphatasia (HPP) is a rare genetic condition caused by deficient activity of the tissue-nonspecific alkaline phosphatase (TNAP), encoded by the *ALPL* gene. In humans, alkaline phosphatase (ALP) activity is also provided by tissue-specific isoenzymes encoded by distinct genes, including intestinal and placental alkaline phosphatases. TNAP hydrolyzes several physiologically important substrates, most notably inorganic pyrophosphate (PPi) and pyridoxal-5′-phosphate (PLP). Accumulation of PPi inhibits hydroxyapatite crystal formation, thereby impairing skeletal mineralization and leading to osteomalacia, fractures, and other mineralization defects (1–3).

Clinically, HPP encompasses a broad spectrum of severity ranging from lethal perinatal forms to mild adult-onset disease, or even asymptomatic cases. Adult HPP is often underdiagnosed or misdiagnosed, owing to its nonspecific manifestations—such as musculoskeletal pain, recurrent stress fractures, or premature tooth loss—and because the hallmark biochemical finding, hypophosphatasemia (low serum ALP), is frequently overlooked in routine practice (4–8).

Besides its involvement in mineralized tissues, TNAP participates in other biochemical pathways, including vitamin B6 metabolism. Normal TNAP activity is needed to hydrolyze pyridoxal-5-phosphate (PLP), a major circulating form of vitamin B6. Consequently, severe TNAP deficiency, particularly in pediatric patients, may result in seizures and other neurological manifestations. Elevated serum PLP levels are commonly observed in HPP and are widely used as a biomarker of TNAP deficiency. Moreover, clinical responsiveness to vitamin B6 supplementation has been proposed as a more reliable diagnostic indicator than baseline PLP concentrations alone (9).

To date, more than 500 pathogenic variants in *ALPL* have been reported, predominantly missense mutations that impair TNAP catalytic activity, intracellular trafficking, or dimerization (10, 11). Persistently low serum ALP remains the biochemical hallmark of HPP. However, even after exclusion of secondary causes of hypophosphatasemia, approximately 40–50% of individuals with low serum ALP do not harbour pathogenic variants in the *ALPL* coding sequence or canonical splicing sites (7, 12–15). The mechanisms underlying reduced ALP activity in these individuals remain unclear. Proposed explanations include undetected genetic alterations such as deep intronic variants, mutations in promoter or regulatory regions, structural rearrangements, or polymorphisms affecting gene expression, as well as variants in other genes influencing *ALPL* regulation (1, 16). Alternatively, epigenetic mechanisms may contribute to reduced *ALPL* expression; notably, DNA methylation of the *ALPL* promoter region has been shown to inversely correlate with gene transcription (17).

TNAP is expressed in multiple tissues, and circulating ALP activity reflects a composite contribution by different isoenzymes (18–21). Accordingly, biochemical characterization of ALP isoenzymes—particularly bone-specific and hepatic fractions—may provide additional insight into the tissue-specific patterns of enzymatic activity. The two major hepatic TNAP isoforms, H1 and H2, as well as bone-derived isoforms, differ primarily in their post-translational modifications.

Despite growing recognition of HPP in clinical practice, integrative studies combining genetic, epigenetic, transcriptomic, and enzymatic analysis in individuals with low serum ALP remain scarce. Consequently, it remains unknown whether genetic, epigenetic, or regulatory mechanisms underlie the reduced enzymatic activity observed in patients with persistent hypophosphatasemia without identifiable *ALPL* mutations. To address this knowledge gap, the present study comprehensively characterizes a cohort of adults with persistently low serum ALP using a multi-omics approach.

## 2 Materials and methods

### 2.1 Study population

We included 50 adult patients with persistently low serum ALP levels (<40 U/L on at least two separate occasions, >6 months apart), sent for evaluation at the Hospital Universitario Marqués de Valdecilla (Santander, Spain). Secondary causes of hypophosphatasemia were systematically excluded by means of a detailed medical history, physical examination, and laboratory tests. In particular, patients with a history of medications known to affect bone metabolism (antiresorptive agents, glucocorticoids, antiepileptics), fibrate use, or other underlying systemic diseases were excluded. The laboratory evaluation included a complete blood count and a biochemical profile comprising albumin, total proteins, transaminases, lactate dehydrogenase, cholesterol, creatinine, sodium, potassium, calcium, phosphate, ALP, vitamin B12, folate, zinc, copper, magnesium, thyroid-stimulating hormone, and parathyroid hormone (1). A control group of 20 healthy adults with normal ALP values, matched by age and sex, was recruited for comparative analysis. The study protocol was approved by the institutional review board (Comité de Ética en Investigation con Medicamentos de Cantabria. Acta 9/2024). All subjects provided informed written consent.

### 2.2 Genetic analysis

#### 2.2.1 *ALPL* promoter region sequencing

Genomic DNA was isolated from 200 µL aliquots of peripheral blood using commercially available column-based extraction kits according to the manufacturers’ instructions. DNA concentration was assessed using both a NanoDrop spectrophotometer and the Qubit dsDNA BR Assay Kit (Thermo Fisher Scientific, Waltham, MA, USA). The proximal promoter region of ALPL (~1,000 bp upstream of the transcription start site, based on the EPDnew annotation for ALPL) was amplified by PCR and analyzed by Sanger sequencing. Primers were designed using Primer3 software (ALPL1_Forward: CGTTCCTAGCTCCGCTCC; ALPL1_Reverse: AGCGTCGAATCCTTACCTGC; ALPL2_Forward: TTCTTCTTGCGGTAGCCAGG; ALPL2_Reverse: TTCCTCTCTCAGCGAGCCT). Sequencing reactions were performed using the BigDye Terminator v3.1 Cycle Sequencing Kit (Applied Biosystems), and products were analyzed on an ABI 3730xl DNA Analyzer.

#### 2.2.2 Whole-exome sequencing

Genomic DNA was initially analysed by targeted sequencing of the ALPL coding exons and flanking intronic regions by NGS. Patients with hypophosphatasemia not carrying ALPL mutations underwent WES using the NextSeq1000 platform (Illumina). Library preparation was performed with Twist (Illumina). Base calling was established with a Q30>90%, and reads were aligned to the GRCh37 reference genome. Sequencing quality met the manufacturer’s specifications, with >95% of target bases covered at ≥40x. The mean sequencing depth across the ALPL coding region was approximately 100x (22).

Variant prioritization was performed using a phenotype-driven strategy implemented in the Franklin platform (Genoox). Human Phenotype Ontology (HPO) terms related to persistent hypophosphatasemia and metabolic bone disease were applied to prioritize variants in genes associated with skeletal mineralization, phosphate metabolism, osteomalacia, skeletal dysplasias, and other inherited disorders potentially explaining persistently low serum alkaline phosphatase levels. Candidate variants were interpreted according to ACMG/AMP recommendations. Variants with a population frequency >5% in gnomAD were excluded. Only coding and splice variants passing quality filters were retained. Variant prioritization incorporated phenotype-driven HPO filtering, inheritance pattern, ClinVar classification, bioinformatic predictors and available literature. Only pathogenic and likely pathogenic variants considered compatible with the patients’ phenotype were retained for interpretation. Variants of uncertain significance without convincing phenotypic correlation were not considered explanatory and are therefore not reported. Copy number variants affecting ALPL were also evaluated using the Canvas CNV caller implemented in the Franklin pipeline.

#### 2.2.3 DNA methylation analysis

Quantitative DNA methylation profiling of the *ALPL* promoter was carried out using the EpiTYPER assay on the MassARRAY system (Agena Bioscience Inc.; San Diego, USA), following the manufacturer’s protocol. EpiTYPER assay is based on the amplification of a region of interest using bisulfite-treated DNA, followed by *in vitro* transcription and RNase digestion. The resulting fragments were analyzed using the MALDI-TOF technology integrated in the MassArray4 system (Agena Bioscience). Aliquots of 1 µg genomic DNA were bisulfite-converted using the EZ DNA Methylation Kit (Zymo Research). PCR primers were designed to cover CpG-rich segments of the promoter and first exon region (chr1:21835620-21836375; GRCh37). All bisulfite-treated DNA samples were analyzed in duplicate, and the methylation percentages were computed using EpiTYPER software v1.2. (Agena Bioscience Inc.; San Diego, USA). Methylation data quality was controlled by removing CpGs with a standard deviation >15% across technical replicates. The average of quantitative DNA methylation values were calculated across informative CpGs.

#### 2.2.4 Gene expression analysis

Total RNA was isolated from whole blood using TRIzol Reagent (Thermo Fisher Scientific), following the manufacturer’s protocol. RNA purity and integrity were assessed using NanoDrop. Reverse transcription was carried out using PrimeScript™ RT reagent kit (Takara), and *ALPL* mRNA levels were quantified by one-step RT-qPCR using TaqMan^®^ Gene Expression Assay (Hs01029144_m1, Thermo Fisher) on an Applied Biosystems 7300 Real-Time PCR System. *ALPL* expression was normalized against two endogenous control genes: *GAPDH* (Hs02786624_g1) and *RPL13A* (Hs04194366_g1). The ΔCt method was applied, and expression levels were calculated as 2^-ΔCt^ for comparative analysis.

### 2.3 Serum total and bone-specific ALP, pyridoxal-5′-phosphate, and bone turnover markers

Fasting blood samples were collected and centrifuged to measure ALP activity in serum. Total ALP was quantified by colorimetric enzymatic assay (pNPP substrate, Atellica^®^ CH Alkaline Phosphatase, Siemens Healthcare Diagnostics, Tarrytown, NY). Intra- and inter-assay coefficients of variation (CV) were <1.5%. Bone-specific ALP (bone ALP) was measured by specific enzyme immunoassay (MicroVueTM BAP EIA, QuidelOrtho Corporation, San Diego, CA, USA). The method is based on the selective capture of bone ALP using a specific antibody, followed by quantification of its enzymatic activity using pNPP as substrate. The antibody exhibits limited cross-reactivity (3–8%) with liver alkaline phosphatase and negligible cross-reactivity with placental or intestinal alkaline phosphatase. Intra- and inter-assay coefficients of variation (CV) were 5.8 and 8% respectively. Serum levels of the bone turnover markers procollagen type I N-terminal propeptide (P1NP) and β-C-terminal telopeptide of type I collagen (β-CTX) were measured using automated chemiluminescence assays (Cobas Pure, Roche Diagnostics, Manheim). The intra-assay CV were 2.3% and 2.2%, respectively, and the inter-assay CV were 3% and 6%, respectively. Pyridoxal-5’- phosphate (PLP) was measured blindly in plasma samples by high-performance liquid chromatography 100 with fluorescence detection (analyzed at Reference Laboratory, Barcelona, Spain, using the kit by Immuchrom, Heppenheim, Germany). The upper limit of normal range is 173 nmol/l; the inter-assay CV is 2.9%.

### 2.4 ALP isoenzyme electrophoresis

ALP isoenzymes were separated and quantified using the HYDRAGEL ISO-PAL K20 kit (Sebia, France), which employs agarose gel electrophoresis followed by visualization with the chromogenic substrate 5-bromo-4-chloro-3-indolyl phosphate/nitro blue tetrazolium in aminomethyl propanol (AMP) buffer (pH 10.1). Serum samples (5 µL) were applied to the gel, and the resolved isoenzymes were subsequently visualized and quantified by densitometric analysis. Separation of hepatic, bone, intestinal, and placental ALP isoenzymes was achieved based on differences in their electrophoretic mobility. The relative contribution of each isoenzyme was determined from the corresponding densitometric peak area. The absolute isoenzyme activity was calculated by multiplying the total serum ALP activity by the proportion represented by each isoenzyme.

### 2.5 Statistical analysis

All data were analyzed using R v4.2. Normality was assessed by Shapiro-Wilk test. Group values were compared by the one-way ANOVA with Tukey’s HSD *post-hoc* test or Kruskal-Wallis for nonparametric data, and Fisher’s exact test for categorical variables. Correlations between continuous variables were assessed using Pearson or Spearman coefficients, as appropriate. A p-value <0.05 was considered statistically significant.

## 3 Results

### 3.1 Genetic characterization and general characteristics of the study cohort

Among 50 adult patients with persistently low serum ALP, sequencing of the *ALPL* gene revealed pathogenic or likely pathogenic variants in 24 individuals (48%). The remaining 26 patients had no identifiable mutations in the *ALPL* coding region. The identified variants were classified as pathogenic or likely pathogenic according to ACMG/AMP criteria and were predominantly heterozygous missense substitutions. Nevertheless, the cohort also included a recurrent heterozygous nonsense variant, c.94C>T, p.(Gln32Ter), detected in two participants, and two heterozygous in-frame deletions, c.1318_1320del, p.(Asn440del), and c.1101_1103del, p.(Ser368del). One participant carried the c.407G>A, p.(Arg136His) variant in homozygosis. The complete list of variants, zygosity, ACMG/AMP classification, and supporting criteria is provided in **Supplementary Table 1**.

Carrier and non-carrier subgroups were of similar age (45 ± 13 yr vs 44 ± 15 yr) and sex distribution (29% vs 20% males). Carriers had a higher prevalence of chronic musculoskeletal pain (78 v.48%, p=0.040), calcific tendinitis (26 vs 4%, p=0.044), and tooth loss (65 vs 32%, p=0.042). Overall, 4% of carriers and 32% of non-carriers did not complain of musculoskeletal symptoms (p=0.024). Among carriers of genetic variants, 65% exhibited high serum PLP levels, whereas none of the non-carriers had serum PLP above the reference range.

Phenotype-driven analysis of the whole-exome sequencing data using Human Phenotype Ontology (HPO)-based filtering did not identify pathogenic or likely pathogenic variants in genes associated with inherited disorders of bone and mineral metabolism or other Mendelian conditions that could plausibly explain persistent hypophosphatasemia in the mutation-negative group. This prompted us to study other levels of gene regulation.

### 3.2 ALPL promoter analysis and gene expression

Targeted Sanger sequencing of the proximal *ALPL* promoter region (~1 kb upstream of the transcription start site) in patients and controls did not identify any sequence variants that could account for the observed differences in ALP activity between groups.

DNA methylation analysis of the same promoter region revealed uniformly low methylation levels across all groups in all 23 CpGs analyzed (<20% mean methylation across CpG sites), consistent with a hypomethylated state compatible with transcriptional permissiveness. No significant differences in methylation patterns were observed between patients and controls.

Consistent with these findings, RT–qPCR analysis of peripheral blood cells demonstrated detectable *ALPL* expression in all individuals, with no statistically significant differences between mutation carriers, mutation-negative patients, and controls (p = 0.134). Moreover, the degree of inter-individual variability in expression was comparable across groups, supporting the presence of active *ALPL* transcription in peripheral blood cells regardless of genetic status.

Taken together, the absence of differences in promoter sequence or methylation status, the lack of identifiable variants in other bone-related genes, and the comparable levels of *ALPL* expression in peripheral blood cells suggest that genomic factors are unlikely to be the primary determinants of the reduced ALP activity observed in hypophosphatasemic patients lacking *ALPL* mutations. Consequently, subsequent analysis focused on the biochemical characterization of circulating enzyme isoforms.

### 3.3 Total and bone-specific ALP

Among individuals with persistent hypophosphatasemia, total serum ALP activity was significantly lower in *ALPL* mutation carriers than in mutation-negative patients (20 ± 5 vs. 32 ± 6 U/L; p < 1 × 10^-6^). As expected, total ALP levels in both patient groups were markedly lower than those observed in controls (56 ± 16 U/L).

Serum levels of the bone turnover markers β-CTX and PINP were significantly reduced in individuals with hypophosphatasemia compared with controls (β-CTX: 0.251 ± 0.146 vs. 0.340 ± 0.220 ng/mL, p = 0.005; PINP: 34.9 ± 14.6 vs. 48.9 ± 24.4 ng/mL, p = 0.0006). However, within individuals with hypophosphatasemia, no significant differences were observed between mutation carriers and non-carriers for either β-CTX (0.261 ± 0.170 vs. 0.235 ± 0.102 ng/mL) or PINP (37.9 ± 15.2 vs. 30.1 ± 12.6 ng/mL).

Consistent with total ALP findings, bone-specific ALP (bone ALP) activity was modestly but significantly lower in mutation carriers than in mutation-negative patients with low total ALP (7.5 vs. 10.1 U/L; p = 0.018), and substantially lower than in controls (19.3 ± 5.9 U/L) (**Figure 1**).

**Figure 1:**
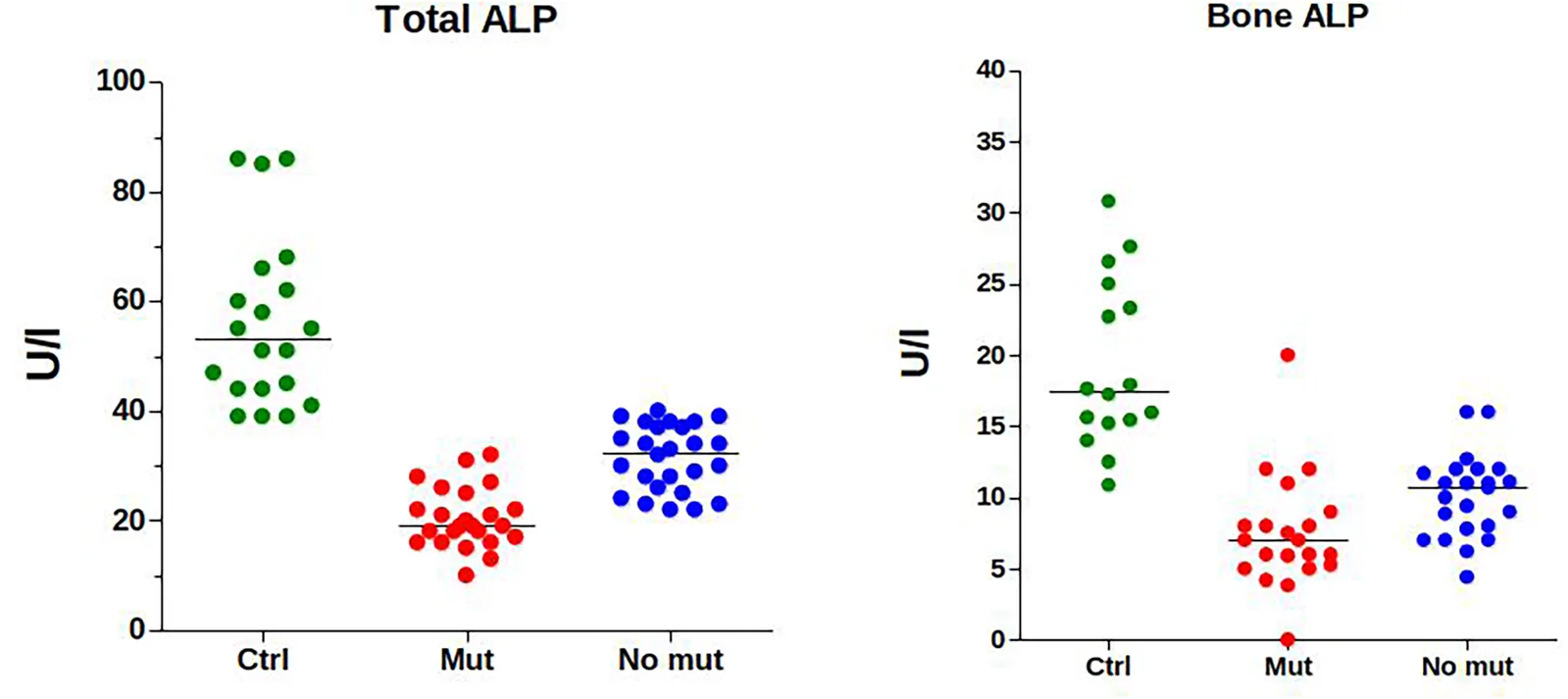
Total ALP levels and bone-specific ALP levels (measured by immunoassay) in the study groups. Horizontal lines correspond to the medians. Ctrl, controls with normal serum ALP; Mut, carriers of ALPL mutations; No mut, individuals with low ALP but without identified mutations. Bone ALP levels of 3 carriers and 3 non-carriers were unavailable. Group values and statistical comparisons are reported in the text as mean ± SD.

In both healthy controls and *ALPL* mutation carriers, total ALP activity showed a significant positive correlation with bone-specific ALP levels (r = 0.78, p = 0.0003 and r = 0.50, p = 0.019, respectively). In contrast, no significant correlation was observed in mutation-negative individuals with low total ALP activity (r = 0.12, p = 0.598), suggesting a dissociation between total and bone-derived ALP activity in this group and potentially reflecting tissue-specific differences between the study groups (**Figure 2**).

**Figure 2:**
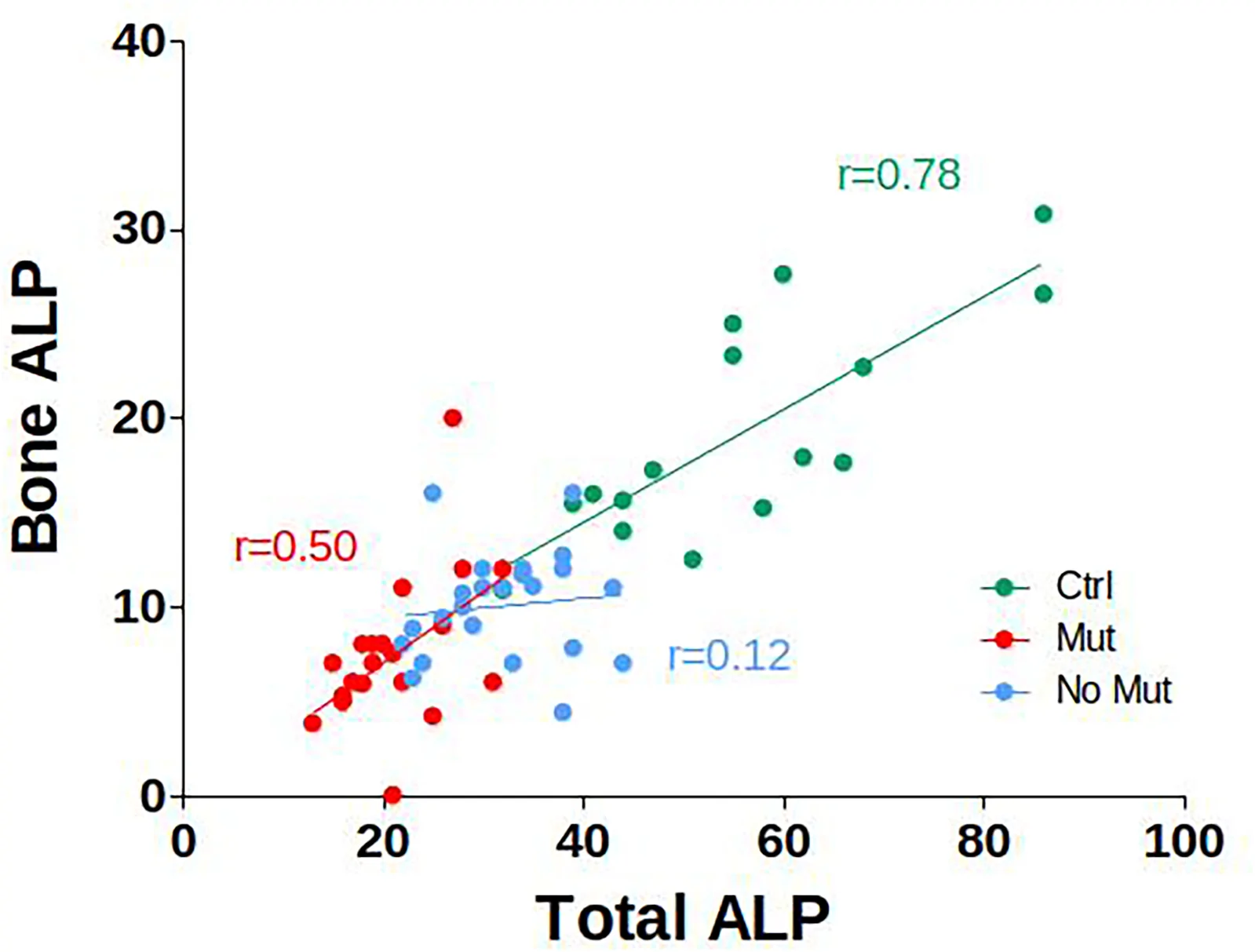
Correlation between total ALP and bone-specific ALP in the three study groups. Ctrl, controls with normal serum ALP; Mut, carriers of ALPL mutations; No mut, individuals with low ALP but without identified mutations.

Based on these observations, we next investigated the relative distribution of circulating ALP isoenzymes.

### 3.4 Isoenzyme electrophoresis profiles

Isoenzyme separation using gel electrophoresis adequately identified the major bone and liver isoforms (H1), as well as the less abundant liver H2 isoform and intestinal isoenzymes.

There was a good correlation between the bone-specific ALP values determined by immunoassay and the results derived from the electrophoresis study (r=0.76 p<10^-11^, **Figure 3**).

**Figure 3:**
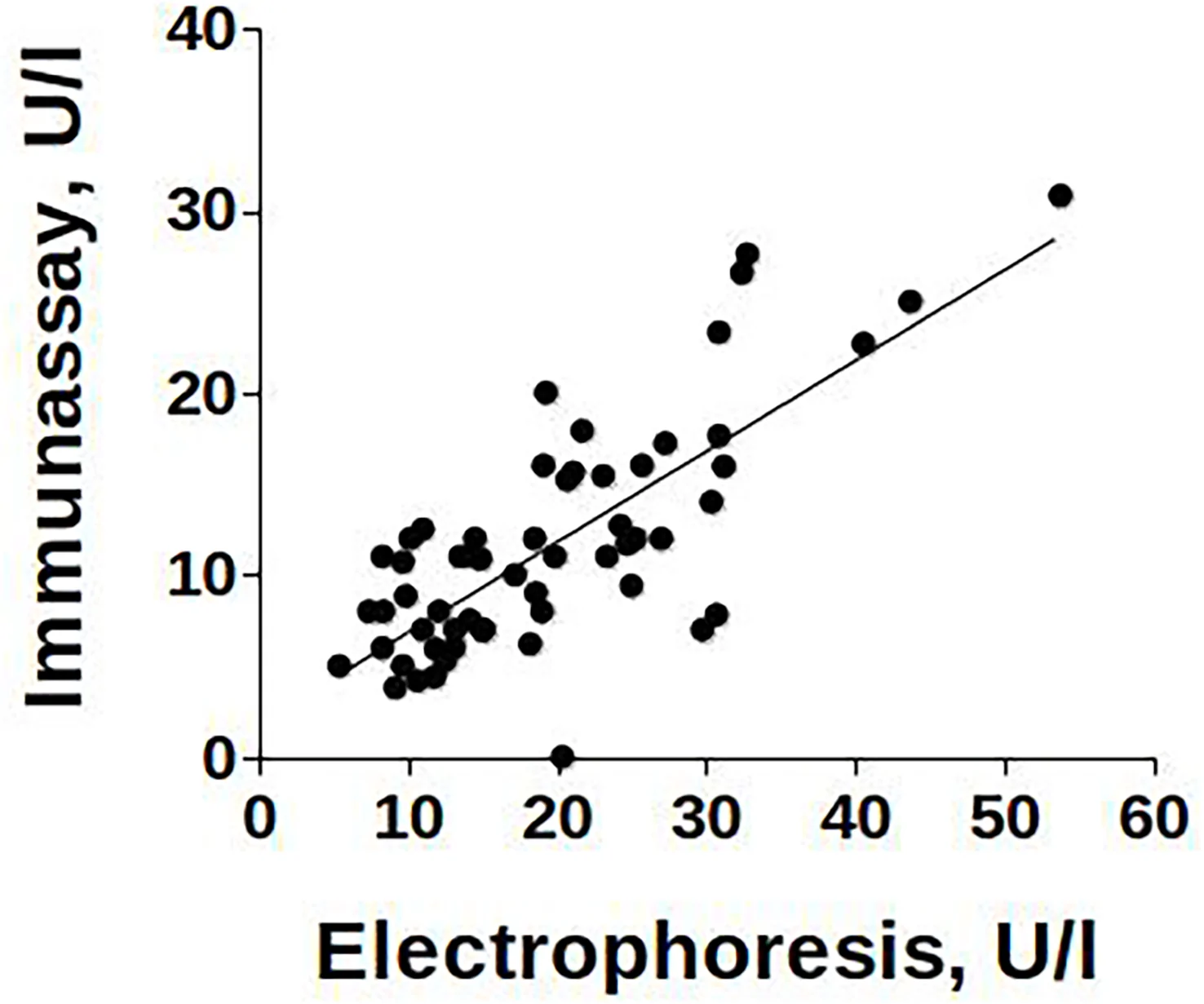
Regression plot of bone-specific ALP as measured by electrophoresis (X axis) and immunoassay (Y axis).

Despite substantial interindividual variability, the relative abundance of the major hepatic isoform (H1) was comparable between controls and mutation-negative patients with low total ALP (40.4 ± 15.3% and 38.5 ± 18.5%, respectively). In contrast, the H1 fraction was significantly reduced in carriers of *ALPL* mutations (26.9 ± 20.2%; p = 0.004 vs. non-carriers).

No significant differences between carriers and non-carriers were observed in the relative proportions of the H2 hepatic isoform (3.6 ± 2.8% vs. 3.1 ± 2.1%), the intestinal isoenzyme (7.8 ± 17.6% vs. 0.3 ± 0.8%), or the bone isoform (61.7 ± 20.6% vs. 56.1 ± 18.8%).

When absolute activities were considered, total hepatic ALP activity—calculated by multiplying total serum ALP activity by the relative abundance of the corresponding isoenzyme—was markedly lower in mutation carriers than in non-carriers (6.1 ± 4.1 vs. 13.9 ± 7.5 U/L; p = 0.0001). The absolute bone isoform activity was also slightly lower in carriers than in non-carriers (12.7 ± 5.0 vs. 18.6 ± 6.5 U/L; p = 0.003; **Figure 4**), and lower than in controls (28.8 ± 10.0 U/L). Thus, the results were consistent with the differences in bone-specific ALP levels measured by immunoassay. The individual bone-to-liver ratio was slightly lower in non-carriers than in carriers, although with substantial variability (1.7 ± 1.1 vs. 2.0 ± 1.3; not significant).

**Figure 4:**
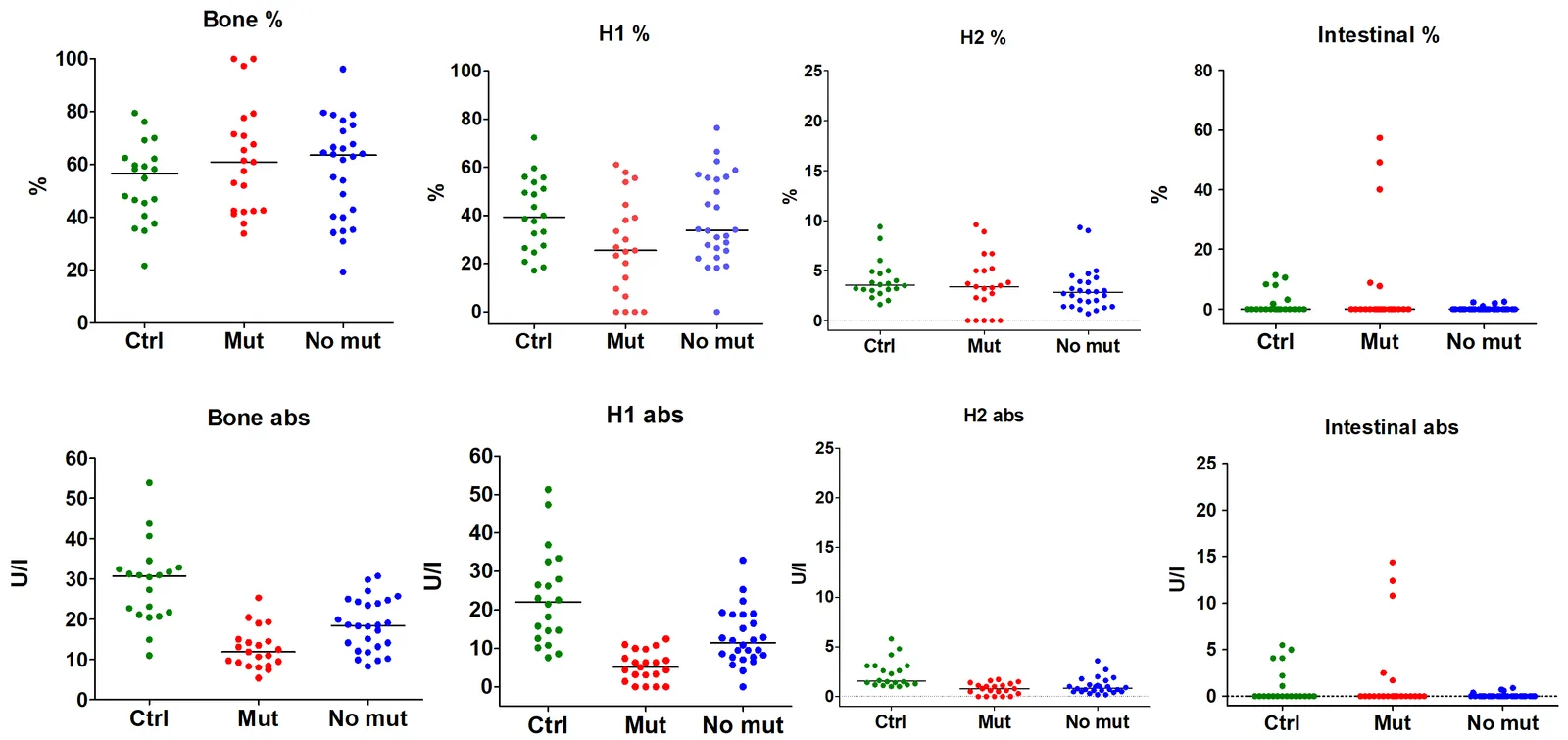
Upper panels: Relative proportion of isoenzymes as measured by gel electrophoresis. Lower panels: Estimated absolute activity of the various isoenzymes. Data unavailable for 3 mutation carriers due to technical problems.

Notably, as summarized in **Figure 5**, the magnitude of ALP reduction in mutation carriers was greater in the hepatic fraction than in the bone fraction. Taken together, these findings suggest that, despite exhibiting overlapping biochemical phenotypes, mutation carriers and non-carriers may develop ALP deficiency through distinct underlying mechanisms.

**Figure 5:**
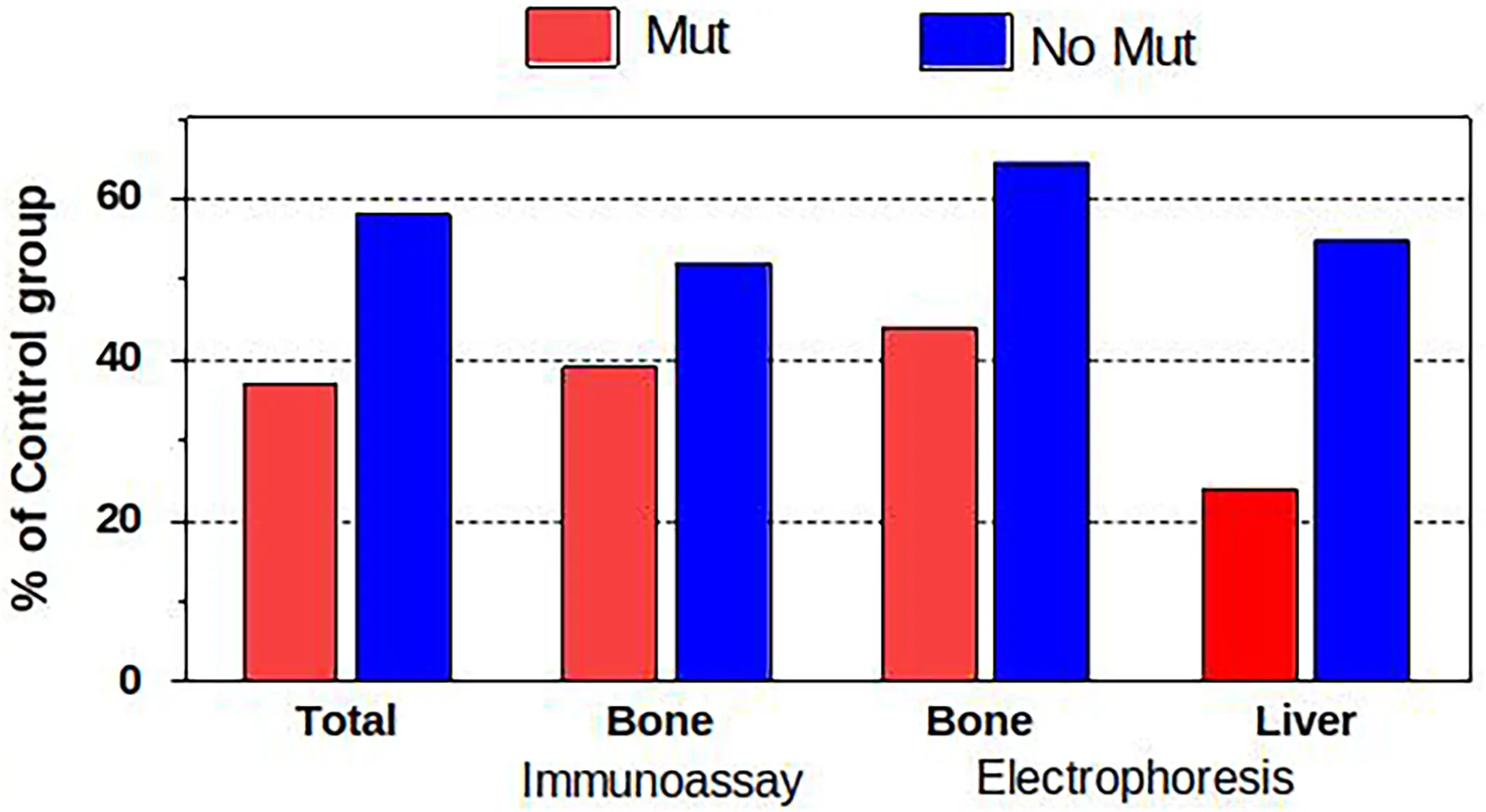
Mean values of total alkaline phosphatase (ALP), bone-specific ALP (measured by immunoassay or gel electrophoresis), and liver fractions (H1 and H2, measured by electrophoresis) in mutation carriers (Mut) and non-carriers (No Mut). Values are expressed as the percentage relative to the mean of the control group.

## 4 Discussion

This study sought to elucidate the mechanisms underlying low serum ALP activity in patients without detectable *ALPL* mutations, who account for roughly one-half of adults with persistently low serum ALP of unknown etiology (12). With that aim, we integrated genomic, epigenomic, transcriptomic, and isoenzymatic data in carriers and non-carriers of pathogenic variants as well as in control subjects.

Contrary to our initial hypothesis that promoter variants might explain reduced ALP expression, targeted Sanger sequencing of the *ALPL* promoter revealed no sequence alterations in either group. Given prior *in vitro* evidence that *ALPL* promoter methylation inversely correlates with gene expression in osteoblastic cells (17), we next evaluated DNA methylation as a potential regulatory mechanism. Promoter methylation analysis demonstrated a consistently hypomethylated state across all individuals, compatible with an open chromatin configuration permissive of gene transcription. These findings argue against transcriptional silencing via CpG hypermethylation as the driver of low ALP activity in non-carriers. Nevertheless, because DNA methylation is highly tissue-specific, we cannot exclude the possibility of epigenetic alterations in hepatocytes or osteoblasts that were not detectable in peripheral blood. Similarly, whole-exome sequencing did not identify single-nucleotide variants (SNVs), indels, or copy number variations (CNVs) in other genes related to mineral metabolism or skeletal homeostasis that could account for the phenotype in *ALPL* mutation-negative individuals.

The preservation of total ALPL mRNA levels in peripheral blood should not be interpreted as evidence against the pathogenicity of the identified variants. Most variants in the mutation-positive group were missense substitutions, which are expected to affect TNAP catalytic activity, folding, dimerization, intracellular trafficking, or protein stability rather than transcription. Furthermore, the cohort included a heterozygous nonsense variant, p.(Gln32Ter), which may lead to nonsense-mediated decay of the mutant transcript. Because the RT-qPCR assay measured total *ALPL* expression without distinguishing between alleles, continued expression from the wild-type allele may mask a reduction in the mutant transcript. In addition, transcript levels in peripheral blood may not reflect allele-specific expression or regulation in osteoblasts and hepatocytes. Thus, comparable *ALPL* mRNA levels across groups do not imply preserved TNAP protein expression or enzymatic function.

Taken together, these negative genomic and epigenomic observations redirect attention from canonical regulatory or coding defects toward tissue-specific differences in enzyme distribution. Indeed, isoenzyme profiling provided further insight: liver ALP activity was relatively preserved in mutation-negative individuals, in contrast to mutation carriers, in whom both liver and bone isoforms were reduced. Moreover, in mutation-negative cases, total ALP levels did not correlate with bone-specific ALP, whereas such a correlation was evident in mutation carriers and controls.

As an index of bone metabolism, bone turnover markers were similarly reduced in both carriers and non-carriers of *ALPL* variants, in agreement with previous reports (23). Notably, low levels of bone turnover markers are a characteristic finding in patients with hypophosphatasia, and the ALP/P1NP ratio has been proposed as a useful tool to distinguish adult HPP from osteoporosis treated with antiresorptive agents (24). In this context, although no overt alterations in bone mineralization have been reported in patients with hypophosphatasemia who lack additional biochemical or genetic features of HPP, these individuals frequently exhibit musculoskeletal complaints similar to those observed in patients carrying *ALPL* mutations and mild adult-onset HPP (25). On the other hand, the notion that liver ALP is not severely affected is supported by previous functional studies from our group, which showed that only individuals carrying *ALPL* variants display an abnormal biochemical response to pyridoxine loading, characterized by accumulation of pyridoxal-5′-phosphate, an ALP substrate predominantly metabolized in the liver (9) (**Figure 6**).

**Figure 6:**
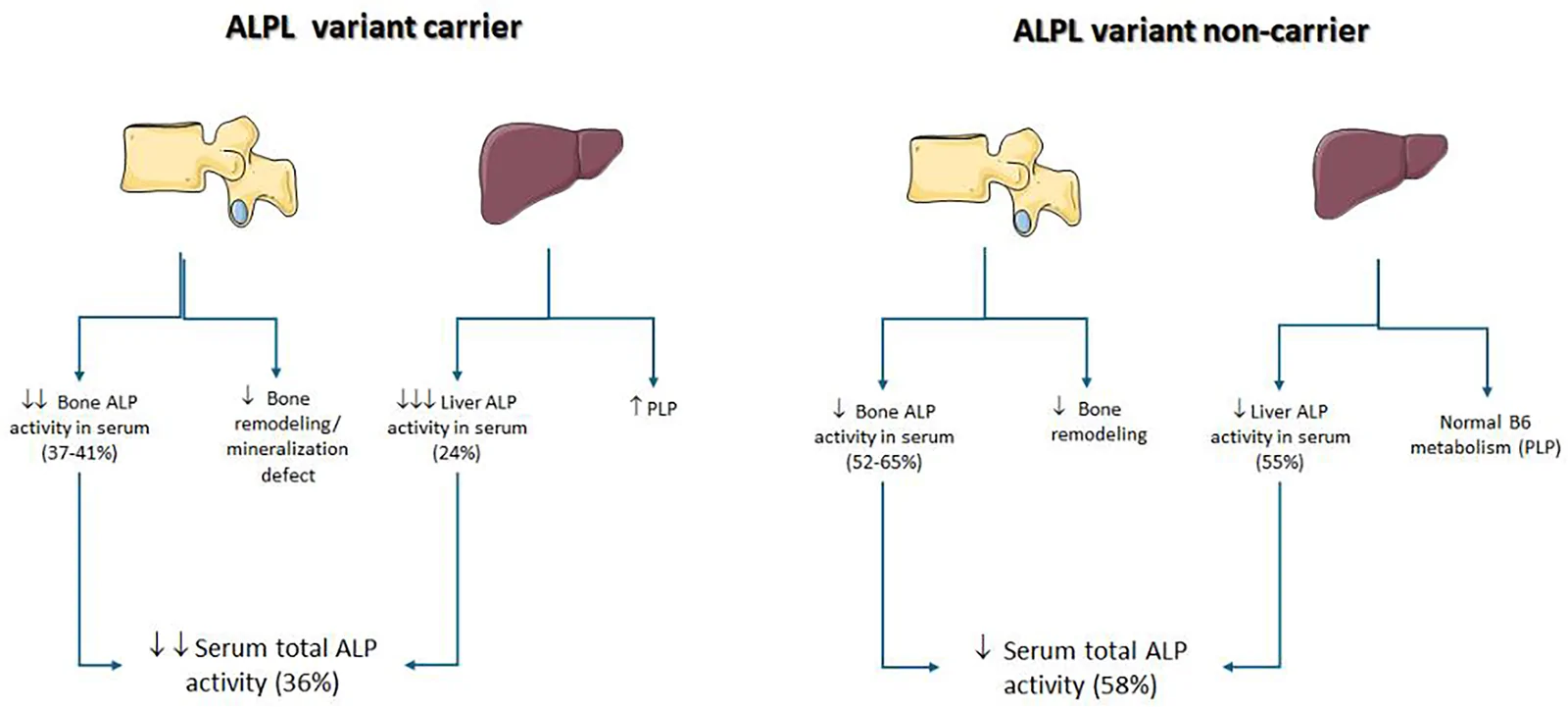
Diagram summarizing ALP changes in carriers and non-carriers of *ALPL* variants, based upon the results of the present study and previous observations. PLP: pyridoxal-5-phosphate. The percentages represent the average enzymatic activity level in comparison with the control group, measured after electrophoretic (bone and liver ALP) and immunocapture (bone ALP). ↓ 25-50% decrease; ↓↓ 50-75% decrease; ↓↓↓ >75% decrease.

There is controversy regarding the diagnosis and nomenclature of individuals carrying pathogenic variants in the *ALPL* gene. Patients presenting with clinical manifestations and biochemical abnormalities characteristic of HPP—namely reduced serum ALP activity and elevated levels of ALP substrates—are confidently diagnosed with “hypophosphatasia”. However, other individuals may exhibit mild or even absent clinical manifestations despite carrying pathogenic *ALPL* variants, together with low serum ALP activity and increased ALP substrates. Some authors classify these individuals as simple “carriers” of a genetic variant. In contrast, we and others (26) propose that they should be considered as having “subclinical hypophosphatasia,” given the presence of clear biochemical abnormalities and the potential for the later development of clinical manifestations. This perspective is consistent with other endocrine and metabolic conditions in which biochemical abnormalities may precede overt clinical disease (for example, “subclinical hypothyroidism”). A third group of individuals, which constitutes the focus of the present study, includes those with persistently low ALP activity in the absence of other underlying disorder, *ALPL* variants and with normal levels of substrates of the TNAP liver isoforms (such as pyridoxal-5′-phosphate). Our data suggest that these individuals may nevertheless exhibit subtle alterations in bone turnover. The long-term clinical significance and trajectory of this biochemical profile remain to be fully elucidated. Therefore, we suggest they should be considered as individuals with “hypophosphatasemia of unknown significance”.

The present study has several limitations. The sample size, although comparable to those in other rare-disease studies, limits the power to detect subtle effects. Methylation was assessed in peripheral blood, which may not fully capture the epigenetic landscape of bone or liver tissues. In fact, DNA methylation patterns are cell- and tissue-specific and may change over time. Unfortunately, practical and ethical considerations precluded the collection of liver and bone samples from these patients. Similarly, potential changes in tissue-specific enhancers were not captured. Although mosaicism is theoretically possible in non-carriers, we are not aware of HPP reports with somatic mosaicism. Regarding isoenzyme analysis, we did not directly estimate the enzymatic activity of each form but relied on the chromatographic separation. Furthermore, while our biochemical assays provide quantitative measures of enzyme activity, they do not directly assess enzyme localization or post-translational regulation.

Clinically, the diagnosis of hypophosphatasia (HPP) often relies on detecting persistently low ALP levels in conjunction with pathogenic *ALPL* variants (3, 27). Our findings suggest that the absence of detectable mutations does not exclude abnormal bone turnover or a hypophosphatasia-like biochemical phenotype (**Figure 6**). Longitudinal follow-up of these patients will be essential to determine whether low ALP levels are associated with long-term musculoskeletal consequences. Future studies incorporating bone and liver tissue analysis will be critical to elucidate the mechanisms driving these abnormalities and to refine diagnostic criteria for mutation-negative cases of low ALP activity.

## Data Availability

Publicly available datasets were analyzed in this study. This data can be found here in ENA under project accession PRJEB128195, which can be accessed directly via the following link: https://www.ebi.ac.uk/ena/browser/view/PRJEB128195.

## References

[1] Riancho JA . Diagnostic approach to patients with low serum alkaline phosphatase. Calcif Tissue Int. (2023) 112:289–96. doi: 10.1007/S00223-022-01039-Y 36348061

[2] Whyte MP . Hypophosphatasia - aetiology, nosology, pathogenesis, diagnosis and treatment. Nat Rev Endocrinol. (2016) 12:233–46. doi: 10.1038/NRENDO.2016.14 26893260

[3] Khan AA Brandi ML Rush ET Ali DS Al-Alwani H Almonaei K . Hypophosphatasia diagnosis: current state of the art and proposed diagnostic criteria for children and adults. Osteoporos Int. (2024) 35:431–8. doi: 10.1007/S00198-023-06844-1 37982857 PMC10866785

[4] Schmidt T Schmidt C Amling M Kramer J Barvencik F . Prevalence of low alkaline phosphatase activity in laboratory assessment: Is hypophosphatasia an underdiagnosed disease? Orphanet J Rare Dis. (2021) 16, 452. doi: 10.1186/S13023-021-02084-W 34711245 PMC8555173

[5] Hepp N Frederiksen AL Duno M Præst Holm J Rye Jørgensen N Beck Jensen J-E . Biochemical, clinical and genetic characteristics in adults with persistent hypophosphatasaemia; Data from an endocrinological outpatient clinic in Denmark. Bone Rep. (2021) 15:101101. doi: 10.1016/j.bonr.2021.101101 34258332 PMC8256181

[6] Schoenau E Neu CM Rauch F Manz F . The development of bone strength at the proximal radius during childhood and adolescence. J Clin Endocrinol Metab. (2001) 86:613–8. doi: 10.1210/jcem.86.2.7186 11158018

[7] Rauch F Bardai G Rockman-Greenberg C . ALPL mutations in adults with rheumatologic disorders and low serum alkaline phosphatase activity. J Bone Miner Metab. (2019) 37:893–9. doi: 10.1007/S00774-019-00991-4 30719581

[8] İnci A Ergin FBC Yüce BT Çiftçi B Demir E Buyan N . Hypophosphatasia: is it an underdiagnosed disease even by expert physicians? J Bone Miner Metab. (2021) 39:598–605. doi: 10.1007/S00774-020-01193-Z 33404770

[9] Alvarez S Ocampo A Caso PC del Real A Puente N Vega AI . Vitamin B6 challenge as a tool for detecting ALPL mutations and diagnosing hypophosphatasia. Osteoporos Int. (2025) 36:1743–7. doi: 10.1007/S00198-025-07595-X 40579471 PMC12460533

[10] Farman MR Rehder C Malli T Rockman-Greenberg C Dahir K Martos-Moreno GÁ . The Global ALPL gene variant classification project: Dedicated to deciphering variants. Bone. (2024) 178:116947. doi: 10.1016/j.bone.2023.116947 37898381

[11] del Angel G Reynders J Negron C Steinbrecher T Mornet E . Large-scale *in vitro* functional testing and novel variant scoring via protein modeling provide insights into alkaline phosphatase activity in hypophosphatasia. Hum Mutat. (2020) 41:1250–62. doi: 10.1002/HUMU.24010 32160374 PMC7317754

[12] Riancho-Zarrabeitia L García-Unzueta M Tenorio JA Gómez-Gerique JA Ruiz Pérez VL Heath KE . Clinical, biochemical and genetic spectrum of low alkaline phosphatase levels in adults. Eur J Intern Med. (2016) 29:40–5. doi: 10.1016/j.ejim.2015.12.019 26783040

[13] Tornero C Navarro-Compán V Tenorio JA García-Carazo S Buño A Monjo I . Can we identify individuals with an ALPL variant in adults with persistent hypophosphatasaemia? Orphanet J Rare Dis. (2020) 15, 51. doi: 10.1186/S13023-020-1315-Y 32066479 PMC7026995

[14] Guarnieri V Sileri F Indirli R Guabello G Longhi M Dito G . Clinical, biochemical and genetic findings in adult patients with chronic hypophosphatasemia. J Endocrinol Invest. (2022) 45:125–37. doi: 10.1007/S40618-021-01625-1 34213743 PMC8741696

[15] Saraff V Narayanan VK Lawson AJ Shaw NJ Preece MA Högler W . A diagnostic algorithm for children with low alkaline phosphatase activities: Lessons learned from laboratory screening for hypophosphatasia. J Pediatr. (2016) 172:181–6. doi: 10.1016/j.jpeds.2016.01.045 26896157

[16] Kishnani PS Rehder C Ozono K Pérez-López J del Angel G Mowrey WR . Revisiting the genetics of hypophosphatasia. J Inherit Metab Dis. (2025) 48(6), e70083. doi: 10.1002/JIMD.70083 41047464 PMC12497681

[17] Delgado-Calle J Sanudo C Sanchez-Verde L Garcia-Renedo RJ Arozamena J Riancho JA . Epigenetic regulation of alkaline phosphatase in human cells of the osteoblastic lineage. Bone. (2011) 49:830–8. doi: 10.1016/j.bone.2011.06.006 21700004

[18] Buchet R Millán JL Magne D . Multisystemic functions of alkaline phosphatases. Methods Mol Biol. (2013) 1053:27–51. doi: 10.1007/978-1-62703-562-0_3 23860646

[19] Millán JL Whyte MP . Alkaline phosphatase and hypophosphatasia. Calcif Tissue Int. (2016) 98:398–416. doi: 10.1007/S00223-015-0079-1 26590809 PMC4824800

[20] Magnusson P Sharp CA Farley JR . Different distributions of human bone alkaline phosphatase isoforms in serum and bone tissue extracts. Clin Chim Acta. (2002) 325:59–70. doi: 10.1016/S0009-8981(02)00248-6 12367767

[21] Minisola S Cipriani C Colangelo L Labbadia G Pepe J Magnusson P . Diagnostic approach to abnormal alkaline phosphatase value. Mayo Clin Proc. (2025) 100:712–28. doi: 10.1016/j.mayocp.2024.11.019 40019430

[22] Richards S Aziz N Bale S Bick D Das S Gastier-Foster J . Standards and guidelines for the interpretation of sequence variants: a joint consensus recommendation of the American College of Medical Genetics and Genomics and the Association for Molecular Pathology. Genet Med. (2015) 17:405–24. doi: 10.1038/GIM.2015.30 25741868 PMC4544753

[23] López-Delgado L Riancho-Zarrabeitia L García-Unzueta MT Tenorio JA García-Hoyos M Lapunzina P . Abnormal bone turnover in individuals with low serum alkaline phosphatase. Osteoporos Int. (2018) 29:2147–50. doi: 10.1007/s00198-018-4571-0 29947871

[24] Bertoldo F Tripepi G Zaninotto M Plebani M Scillitani A Varenna M . Possible role of bone turnover markers in the diagnosis of adult hypophosphatasia. J Bone Miner Res. (2024) 40:79–86. doi: 10.1093/JBMR/ZJAE177 39498489 PMC11983269

[25] Santurtun M Mediavilla-Martinez E Vega AI Gallego N Heath K Tenorio JA . Pain and health-related quality of life in patients with hypophosphatasemia with and without ALPL gene mutations. Front Endocrinol (Lausanne). (2022) 13:965476. doi: 10.3389/fendo.2022.965476 36072928 PMC9442670

[26] Seefried L Genest F Hofmann C Brandi ML Rush E . Diagnosis and treatment of hypophosphatasia. Calcif Tissue Int. (2025) 116, 46. doi: 10.1007/S00223-025-01356-Y 40047955 PMC11885340

[27] Khan AA Josse R Kannu P Villeneuve J Paul T Van Uum S . Hypophosphatasia: Canadian update on diagnosis and management. Osteoporos Int. (2019) 30:1713–22. doi: 10.1007/S00198-019-04921-Y 30915507

