# Supplementary material for: Multilevel analysis of patients with hypophosphatasemia: genomic, epigenomic and isoenzyme activity profiles
Source: Front Endocrinol (Lausanne). 2026 Oct 30;17:1910750. doi: 10.3389/fendo.2026.1910750 (PMC13759390; doi:10.3389/fendo.2026.1910750)
Supplement: Supplementary file 1 [file Table1.docx]

**Supplementary Table 1. ALPL variants identified in patients with persistent hypophosphatasemia**

| **Variant** | **ALPL cDNA variant*** | **Protein consequence** | **Zygosity** | **Molecular consequence** | **ACMG/AMP classification** |
| --- | --- | --- | --- | --- | --- |
| V01 | c.407G>A | p.(Arg136His) | Homozygous | Missense | Pathogenic |
| V02** | c.1426G>A | p.(Glu476Lys) | Heterozygous | Missense | Pathogenic |
| V03 | c.1318_1320delAAC | p.(Asn440del) | Heterozygous | In-frame deletion | Pathogenic |
| V04** | c.1418G>A | p.(Gly473Asp) | Heterozygous | Missense | Pathogenic |
| V05** | c.200T>C | p.(Val67Ala) | Heterozygous | Missense | Likely pathogenic |
| V06 | c.299C>T | p.(Thr100Met) | Heterozygous | Missense | Pathogenic |
| V07** | c.94C>T | p.(Gln32Ter) | Heterozygous | Nonsense | Pathogenic |
| V08 | c.382G>A | p.(Val128Met) | Heterozygous | Missense | Pathogenic |
| V09** | c.334G>C | p.(Gly112Ser) | Heterozygous | Missense | Pathogenic |
| V10 | c.247G>A | p.(Glu83Lys) | Heterozygous | Missense | Likely pathogenic |
| V11 | c.454C>T | p.(Arg152Cys) | Heterozygous | Missense | Pathogenic |
| V12 | c.353T>G | p.(Leu118Arg) | Heterozygous | Missense | Likely pathogenic |
| V13 | c.659G>T | p.(Gly220Val) | Heterozygous | Missense | Pathogenic |
| V14** | c.497C>T | p.(Thr166Ile) | Heterozygous | Missense | Pathogenic |
| V15 | c.443C>T | p.(Thr148Ile) | Heterozygous | Missense | Pathogenic |
| V16 | c.1101_1103delCTC | p.(Ser368del) | Heterozygous | In-frame deletion | Pathogenic |
| V17 | c.1366G>A | p.(Gly456Arg) | Heterozygous | Missense | Pathogenic |

* Variants are reported relative to the ALPL reference transcript used in the genetic analysis: NM_000478.6.

** Variants identified in at least two individuals.
